# Microplastic pollution in the sediments of interconnected lakebed, seabed, and seashore aquatic environments: polymer-specific total mass through the multianalytical “PISA” procedure

**DOI:** 10.1007/s00216-023-04664-0

**Published:** 2023-04-18

**Authors:** Andrea Corti, Jacopo La Nasa, Greta Biale, Alessio Ceccarini, Antonella Manariti, Filippo Petri, Francesca Modugno, Valter Castelvetro

**Affiliations:** 1 Department of Chemistry and Industrial Chemistry, University of Pisa, 56124 Pisa, Italy; 2 CISUP - Center for the Integration of Scientific Instruments of the University of Pisa, University of Pisa, 56124 Pisa, Italy

**Keywords:** Polyolefin, Polystyrene, PET, Nylons, PVC, Pyrolysis-GC/MS

## Abstract

The total mass of individual synthetic polymers present as microplastic (MP < 2 mm) pollutants in the sediments of interconnected aquatic environments was determined adopting the Polymer Identification and Specific Analysis (PISA) procedure. The investigated area includes a coastal lakebed (Massaciuccoli), a coastal seabed (Serchio River estuarine), and a sandy beach (Lecciona), all within a natural park area in Tuscany (Italy). Polyolefins, poly(styrene) (PS), poly(vinyl chloride) (PVC), polycarbonate (PC), poly(ethylene terephthalate) (PET), and the polyamides poly(caprolactame) (Nylon 6) and poly(hexamethylene adipamide) (Nylon 6,6) were fractionated and quantified through a sequence of selective solvent extractions followed by either analytical pyrolysis or reversed-phase HPLC analysis of the products of hydrolytic depolymerizations under acidic and alkaline conditions. The highest concentrations of polyolefins (highly degraded, up to 864 µg/kg of dry sediment) and PS (up to 1138 µg/kg) MPs were found in the beach dune sector, where larger plastic debris are not removed by the cyclic swash action and are thus prone to further aging and fragmentation. Surprisingly, low concentrations of less degraded polyolefins (around 30 µg/kg) were found throughout the transect zones of the beach. Positive correlation was found between polar polymers (PVC, PC) and phthalates, most likely absorbed from polluted environments. PET and nylons above their respective LOQ values were found in the lakebed and estuarine seabed hot spots. The pollution levels suggest a significant contribution from riverine and canalized surface waters collecting urban (treated) wastewaters and waters from Serchio River and the much larger Arno River aquifers, characterized by a high anthropogenic pressure.

**Graphical abstract:**

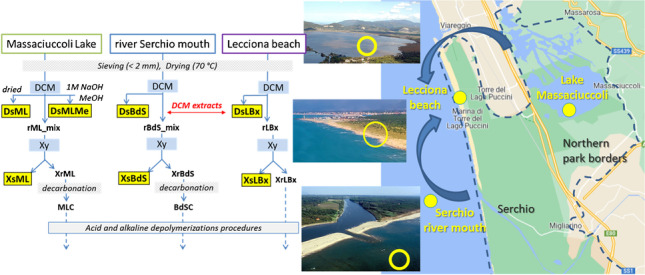

**Supplementary Information:**

The online version contains supplementary material available at 10.1007/s00216-023-04664-0.

## Introduction

The nearly ubiquitous presence of polymeric particulate pollutants, commonly referred to as microplastics (MPs), in both aquatic and terrestrial environments has been recognized to be mainly originated by land-based human activities including agriculture (mulching films and other plastic items that are often dispersed in the environment) [1, 2], industrial production and industrial products (plastic pellets, secondary particles from fragmentation of larger plastic items, coatings, and tire wear) [3-5], and households (synthetic textile fibers, microplastics from home care and personal care formulations, etc.) [6, 7]. These materials, once dispersed in the environment and typically entering water bodies, undergo progressive degradation while being transported until settling in sediments as the most likely ultimate sink [8, 9]. Once activated, their environmental degradation proceeds with chemical (oxidation, polymer chain fragmentation) and physical (fragmentation down to the nanometric size) degradation to molecular species [10, 11]. The rate of such processes is dependent on the chemical nature of the polymer and on the exposure to photo-oxidizing conditions, the latter being also influenced by possible recirculation between benthic sediment and the water column through animal ingestion and biofouling [12, 13]. The evaluation of possible correlations among the types and amounts of MPs in different but interconnected aquatic environments, such as those collecting surface waters from regions with anthropogenic pressure deriving from a mix of urban, agricultural, and industrial activities, is thus of particular interest.

In the present study, the MPs content of sediment samples from three interconnected aquatic habitats within the protected natural area of the natural park Migliarino San Rossore Massaciuccoli, namely a shallow coastal lake (Massaciuccoli), a coastal seabed (Serchio River estuarine), and a sandy beach (Lecciona), was measured. The park area stretches about 30 km along the northern coast of Tuscany, Italy, with the estuary of river Arno (its aquifer including most of the northern Tuscany region with over 2 million inhabitants) as the southern border and the touristic town of Viareggio as the northern one. It is characterized by a surrounding high anthropogenic pressure, with agricultural, industrial, urban, and touristic activities as potential sources of MP pollution. In this context, the seabed, the lakebed, and the marine beach are typical sites of accumulation of microplastics. The latter are made of different polymers, their different densities resulting in a tendency to float or sink in freshwater and seawater that is also dependent on their formulation (foamed, with or without inorganic fillers, etc.). A previous report on the pollution by microplastics in the sandy beach area of the natural park environment close to its southernmost border, sandwiched between the estuaries of Serchio and Arno rivers, was not intended to assess the actual concentration of pollutants but rather the type of materials polluting an unmanaged and restricted area, where plastic items and MPs can only come from the sea and the nearby river mouth [14]. Similarly, previous papers focused on the sandy beach of Marina di Vecchiano, located at the mouth of the Serchio River, were either limited to hydrocarbon polymers (polyolefins and PS) [15] or mainly intended at developing a procedure for polymer-specific MP mass quantification [16, 17] as opposed to the more conventional separation and counting approach, the latter providing only semi-quantitative data on the pollution load by MPs in a given environment.

As for the analysis of MPs, the most commonly used spectroscopic (mainly Fourier transform infrared micro-spectroscopy, μ-FTIR, and μ-Raman) and thermoanalytical (pyrolysis-gas chromatography-mass spectrometry, Py-GC/MS, and thermal extraction-desorption-GC-MS, TED-GC-MS) techniques have been exhaustively reviewed [18]. The former give particle-based information regarding the polymer type, number, size, distribution, and shape, with advanced data processing tools being actively developed [19, 20], but with detection typically limited at particles above 10∼20 μm. The latter provide mass-based information, allowing the obtaining of quali-quantitative information not only on MPs but also on associated low molecular weight species such as polymer additives and other substances captured by the MPs from contaminated environmental matrices or released from slowly degrading polymers [21–24].

In this work, the total mass of individual synthetic polymers present as microplastic (MP < 2 mm) pollutants in the sediments was determined by adopting the Polymer Identification and Specific Analysis (PISA) procedure [25]. In particular, the following polymers were targeted: polyolefins (polypropylene (PP) and polyethylenes (PE), including both high-density (HDPE) and low-density (LDPE)) and poly(styrene) (PS), all of which are characterized by density lower than that of seawater unless heavily oxidized or biofouled, and the comparatively higher-density poly(vinyl chloride) (PVC), polycarbonate (PC), poly(ethylene terephthalate) (PET), poly(6-aminohexanoic acid) (Nylon 6), and poly(hexamethylene adipamide) (Nylon 6,6). The PISA procedure allows individually quantifying such main polymer types through a sequence of selective solvent extractions followed by Py-GC/MS [20], and of hydrolytic (acidic for polyamides [26], alkaline for polyesters [27]) depolymerizations followed by reversed-phase HPLC analysis to quantify the obtained free monomers.

## Materials and methods

### Environmental samples

Sediment samples were collected from Lecciona beach (LB), Lake Massaciuccoli (LM), and the nearby estuarine continental platform of Serchio River (BdS), all located about 10–15 km north of the mouth of river Arno in Tuscany, Italy, and belonging to the Regional Natural Park of Migliarino, San Rossore, and Massaciuccoli facing the Thyrrhenian Sea. Lake Massaciuccoli is a shallow coastal lake connected to the sea through a canal with locks preventing tidal saltwater intrusion. The nearby marine beach of Lecciona, located halfway between the touristic beaches of Torre del Lago and Viareggio, is an unmanaged sandy coastal area characterized by a stretch of back dunes with rich vegetation; as the seashore is accessible to tourists from the surrounding touristic beaches, it undergoes cleaning twice a year. Serchio River runs across the Garfagnana valley and the densely populated and industrialized province of Lucca.

Beach sand samples were collected on May 26, 2020 (that is, just after a 2-month total lockdown in Italy because of the pandemic, and therefore without any significant recent contamination from beach tourism), from dune, winter berm, summer berm, and foreshore, respectively, the sampling points being spaced apart about 8–10 m within the transect (depending on the shore morphology). Three cross-shore transects about 50 m from each other were sampled using cylindrical glass bottles as corers to collect the upper 5-cm layer of the sand (samples LBd1-3, LBw1-3, LBs1-3, LBf1-3). A van Veen grab sampler was used for fine sandy marine sediments at about 5 m depth from three nearby points about 500 m off the Serchio River mouth (samples BdS1-3) on May 8, 2022. Finally, an Ekman box corer was used to collect silty sediment at about 2 m depth from three nearby points in Lake Massaciuccoli (samples ML1-3) on May 3, 2022, close to the entrance into the lake of one of the main canals (Fosso della Barra) conveying waters from the surrounding agricultural plane, from a wastewater treatment plant serving the small town of Vecchiano, and from the intermittent seasonal creeks running down the nearby Pisan Mountains. A georeferenced list of all the samples analyzed and the wet and dry weights of the samples are listed in figure S1 and table S1 in the Supplementary information (SI). The sediment samples were sieved at 5.0 mm followed by sieving at 2.0 mm to fractionate microplastic, mesoplastic, and other material of biogenic origin. The 2–5-mm synthetic polymer fragments were manually separated from biogenic and inorganic debris with the help of a stereoscopic microscope (magnification 10–40×). The sieved fraction < 2 mm was stored refrigerated in glass bottles with metal screw caps before drying at 70 °C in a ventilated oven to constant weight (sediment placed in glass or metal trays with punctured aluminum foil covers to prevent contamination from airborne microplastics) followed by mechanical homogenization of the typically uniform sediment (very fine for the ML and BdS samples, a bit coarser for the LB ones) with a spoon in a metal tray; all operations were performed in a clean fume hood by operators wearing cotton coats, and the sediment samples were then stored in glass jars with metal screw caps before use. Mass concentrations of microplastics are referred to the dry weight of the sediment.

### Chemicals and materials

Dichloromethane (DCM), xylene (Xy), and methanol (all HPLC grade, Sigma-Aldrich) were used as solvents for sequential boiling solvent extraction and solvent-antisolvent precipitation of the extracted polymers, when specified. Hydrochloric acid (ACS reagent, 37%, Sigma-Aldrich) and sodium hydroxide (ACS reagent, ≥ 97.0%, pellets, Sigma-Aldrich) were used as received or diluted with Milli-Q™ water produced with a Millipore Milli-Q Water Purification System. Five reference polymers were used to optimize the operating conditions of the pyrolysis-gas chromatography-mass spectrometry (Py-GC/MS) equipment and obtain calibration curves for semi-quantitative analysis of the solvent extracts: polystyrene (PS), high-density polyethylene (HDPE), and polypropylene (PP) additive-free micropowders produced by cryomilling were a kind gift of Poliplast SpA (Casnigo, Italy); poly(vinyl chloride) (PVC) was scraped off a commercial pipe and analyzed by FTIR and soxhlet extraction with methanol to ascertain the absence of plasticizers (typically not employed for high-resistance PVC pipes); polycarbonate (PC) was a pure product from Sigma-Aldrich. Dimethyl phthalate, dibutyl phthalate, benzyl-butyl phthalate, bis(2-ethylhexyl) phthalate bis(7-methyloctyl) phthalate, and bis(8-methylnonyl) phthalate (analytical standard grade, Sigma-Aldrich) were used as reference species to validate the method and obtain calibration curves for plasticizers, while dibutyl phthalate-3,4,5,6-d^4^ (DS1) and dioctyl phthalate-3,4,5,6-d^4^ (DS2) (analytical standard grade, Sigma-Aldrich) were used as internal standards.

### Analytical procedures, quality assurance, and quality control (QA/QC) of the quantitative results

The polymer-specific total mass of each synthetic polymer type present as microplastic pollutants in each sediment sample was determined by performing the Polymer Identification and Sequential Analysis (PISA) procedure [25]. The targeted polymers were polyolefins (low-density polyethylene (LDPE) along with HDPE and PP), PS, PVC, PC, poly(ethylene terephthalate) (PET), poly(6-aminohexanoic acid) (Nylon 6), and poly(hexamethylene adipamide) (Nylon 6,6), where PC and PVC represent an extension of the cited PISA procedure, as specified in section “Pyrolysis-gas chromatography-mass spectrometry (Py-GC/MS).” The PISA procedure consists in subsequent fractionation steps based on selective extraction in boiling solvents (DCM and Xy), followed by treatments of the sediment residue under acid and alkaline hydrolysis conditions, respectively, to achieve total depolymerization of all polyamides and polyesters, respectively. The synthetic polymers isolated in the various fractions are then quantified by using suitable chromatographic characterization techniques as outlined below.

All solvent extraction, hydrolysis, and analytical characterizations have been validated through QA/QC procedures. In detail, the extractions with refluxing solvent were routinely carried out in triplicate on sediment samples mechanically homogenized with a spoon on a metal tray, and procedural blanks were performed by running solvent extractions with the same apparatus loaded with empty cellulose thimbles, same amounts of solvent, and same extraction times as those used to extract the sediment samples. Procedural blanks have also been run for the solvent concentration and polymer precipitation procedure in the case of xylene extracts, and for the alkaline and acid hydrolysis, respectively, in the pre-concentration phase on SPE C18 columns used for terephthalic acid (TPA) analysis (see section “Alkaline depolymerization: determination of PET”), and in the derivatization with dansyl chloride (DNSCl) of 6-aminohexanoic acid (AHA) and hexamethylene diamine (HMDA) amino monomers (see section “Acid depolymerization: determination of Nylon 6 and Nylon 6,6 polyamides”). The depolymerization and HPLC analyses were run in triplicate for the beach sediment samples, while the amount of available sediment was not sufficient for triplicate analyses on the LM and BdS samples. Each run of HPLC analysis was followed by a blank run (injection of the pure solvent) to exclude any potential memory effect or erratic peaks in the chromatographic column. Recovery rates always above 95% were measured by running extraction or depolymerization/HPLC analyses on spiked sediment samples.

**Fig. 1:**
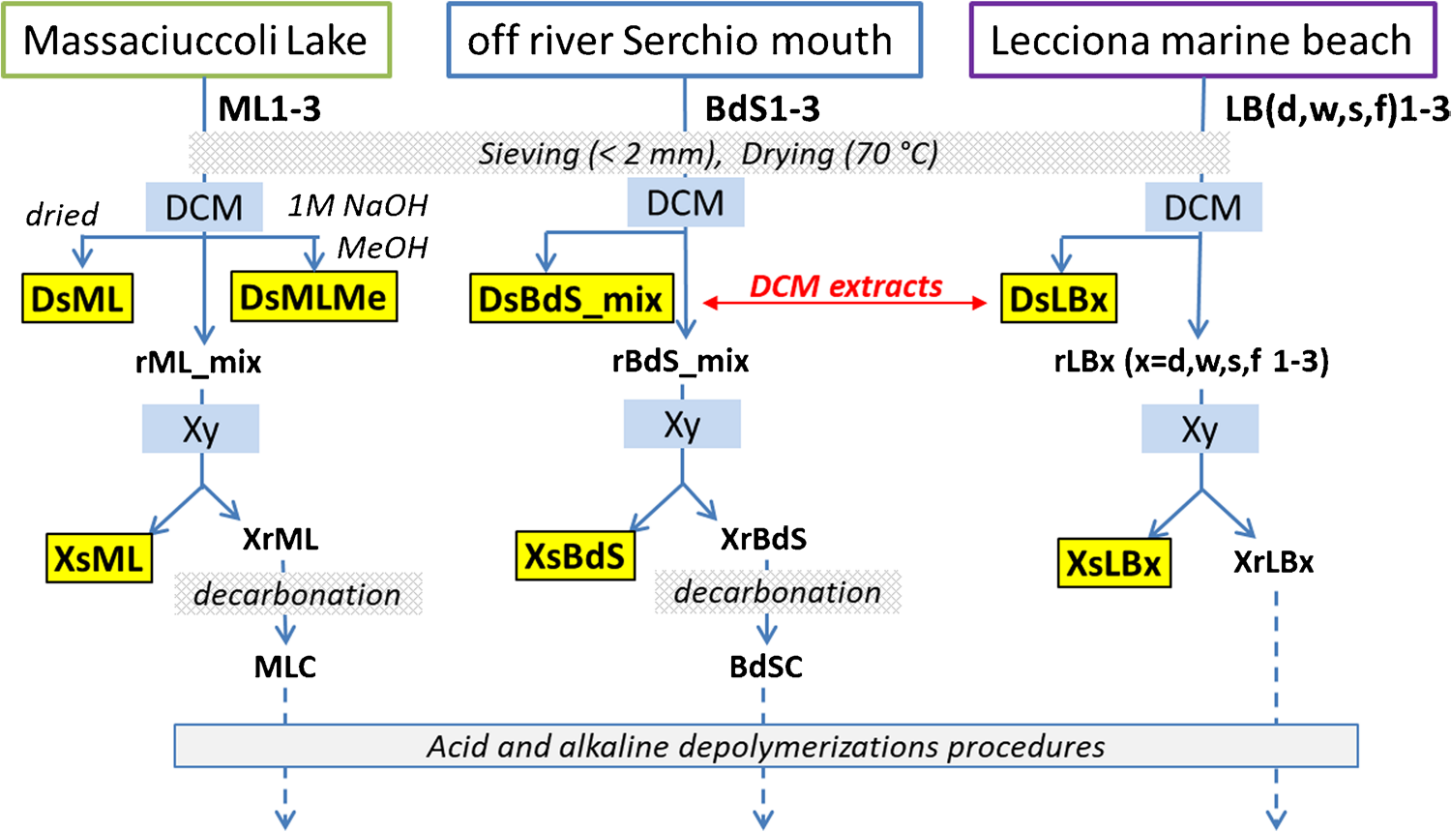
General scheme of the sequential solvent extraction steps, corresponding to the first two steps of the PISA analytical protocol, as adapted for the presence of excess biogenic material or carbonates. The highlighted labels indicate the final samples that were analyzed for polymer content, while the dashed arrows refer to the subsequent treatments for the quantification of PET and polyamides

The overall sample treatment and separation procedure as outlined in Fig. 1 is described in detail in the following sub-sections. The labels used for the various fractions are based on the following keys: BdS, ML, and LB are the sampling sites (see section “Environmental samples”); Ds and Xs are the fractions extracted with DCM and Xy, respectively (so DsML stays for the DCM-extractable fraction from a ML sediment); r as a prefix indicates the insoluble residue after DCM extraction, while Xr is the insoluble residue after extraction with Xy; other specific keys are explained in the detailed descriptions of sections “Extraction in refluxing dichloromethane (DCM)” to “Acid depolymerization: determination of Nylon 6 and Nylon 6,6 polyamides.”

#### Extraction in refluxing dichloromethane (DCM)

In the first step, each dried sediment sample placed in a cellulose thimble was extracted for 6 h in refluxing DCM (typically 200 mL solvent for 50 g sediment) using either a Kumagawa apparatus or an automatic Büchi E-800 hot solvent extractor. The extractable fraction may typically contain PS, low molecular weight polyolefin fragments with a high degree of oxidation [17], PC, and most vinyl polymers such as, e.g., PVC and acrylics, along with biogenic material (mainly lipids). Once the system was cooled, the DCM extract was concentrated at reduced pressure before undergoing analytical characterizations.

However, a partial modification of the general procedure was adopted in the case of sediment samples from Lake Massaciuccoli (ML1, ML2, and ML3) presumably characterized by a very high content of organic matter of plant origin. In particular, the DCM extracts obtained from the individual samples were combined into a single solution, which was then divided into two fractions of equal volume. The first DCM fraction, hereinafter referred to as DsML (see Fig. 1), was evaporated to dryness in the same way as all other samples. The second DCM fraction was extracted twice with aqueous 1 M NaOH in a separating funnel to remove most interferents such as low molecular weight carboxylic acids of biogenic origin. After separation, the organic fraction was dried on anhydrous sodium sulfate, concentrated at reduced pressure, and added dropwise to a 20-fold excess MeOH to precipitate polystyrene (PS) and any medium–low molecular weight oxidized (highly degraded) polyolefins. The precipitated solid was recovered by suction filtration on a 0.22-μm-pore-size PVDF filter membrane (sample DsMLMe).

#### Extraction in refluxing xylene

The sediment samples previously extracted with DCM were subjected to a second extraction with refluxing xylene for 5 h to collect any high molecular weight polyolefins. In particular, the solid residues of the DCM extraction of the marine beach sediment samples of Lecciona (rLBx, with *x* = *d*, *w*, *s*, *f*) were treated individually, while those from the three samples ML1-3 and from the three samples BdS1-3 were combined into the medium composites rML_mix and rBdS_mix, respectively, before performing the subsequent step of extraction with refluxing xylene. At the end of the extraction, the solution was concentrated to about 1/8 of the initial volume by removing the xylene by distillation directly from the Kumagawa extractor. The concentrated solution, once cooled to about 50 °C, was added to a 20-fold volume excess of methanol to promote the precipitation of medium-to-high molecular weight polyolefins. The precipitated solids were recovered by suction filtration on a pre-weighed 0.22-μm-pore-size PVDF filter membrane (samples XsML, XsBdS, XsLBx) and analyzed by ATR spectroscopy directly on the filter. For the subsequent analysis by Py-GC/MS, the polymer was retrieved from the filter by soaking in ethanol and applying mild sonication with an ultrasonic bath for 30 min, then drying with a nitrogen purge.

#### Sediment sample pre-treatment for hydrolytic depolymerizations

A preliminary inspection had shown the presence of large fractions of carbonates in the solvent-extracted sediment samples of Lake Massaciuccoli and the Serchio River mouth, which could interfere with the acid hydrolysis step required for the quantitative depolymerization of polyamides. Therefore, a pre-treatment was carried out to remove carbonates from the residues XrML and XrBdS of the xylene extraction performed on rML_mix and rBdS_mix, respectively. Thus, XrML and XrBdS were added dropwise with 1 M HCl at room temperature, periodically checking the pH until complete evolution of gases resulting from the hydrolytic conversion of the inorganic carbonates into CO_2_ and soluble chlorides (CaCl_2_). The sediment residue was then filtered on a 0.22-μm PVDF membrane, washed with distilled water, and dried in a ventilated oven at 70 °C until constant weight. The resulting decarbonated sediment samples were divided into two fractions of equal mass, MLC1 and MLC2, BdSC1 and BdSC2, to perform independently the alkaline and acid hydrolysis steps for the depolymerization of polyamides and PET, respectively. Again, the residues XrLBx (*x* = *d*, *w*, *s*, *f*) from the xylene extraction of the samples from Lecciona were individually treated as such to both acid and alkaline hydrolysis.

#### Alkaline depolymerization: determination of PET

For the quantification of the PET content, the residues XrLBx, MLC1, and BdSC1 from the xylene extraction were treated under alkaline conditions suitable for inducing total hydrolytic depolymerization to the corresponding co-monomers terephthalic acid (TPA) and ethylene glycol [27]. To this end, each sample was mechanically stirred for 8 h in aqueous 2 M NaOH at 85 °C, in the presence of catalytic amounts of tributyl-hexadecylphosphonium bromide (TBHDPB) as a phase transfer catalyst. At the end of the reaction, the mixtures were cooled to room temperature and filtered through a 0.22-μm-pore-size PVDF filter. For the removal of excess organic contaminants before HPLC analysis, 1 mL of alkaline hydrolysate was weighed at 0.1 mg accuracy and treated in a 50-mL Erlenmeyer flask with 1–2 mL of 30 vol% H_2_O_2_ until complete discoloration and/or end of visible bubble formation, then 1 mL 1.9 M H_2_SO_4_ was added to convert the terephthalic salt in the neutral TPA. The resulting acidic solution was eluted through a reversed-phase SPE cartridge (Macherey-Nagel Chromabond® C18ec, loaded with 500 mg stationary phase), then the adsorbate was desorbed with 0.8 mL MeOH and the recovered roughly 0.8 mL solution in methanol was weighed at 0.1 mg accuracy. Finally, 0.5 mL of the solution was taken up with a micropipette, placed in a vial, and weighed again at 0.1 mg accuracy, then added with 0.75 mL aqueous trifluoroacetic acid (0.1 wt% in HPLC-grade water) to obtain a 40/60 vol% methanol/water mixture. TPA quantification was then performed by injecting this mixture in a 50-μL loop of the reversed-phase HPLC instrument, as described in sections “High-performance liquid chromatography (HPLC).”

#### Acid depolymerization: determination of Nylon 6 and Nylon 6,6 polyamides

For the quantification of Nylon 6 and Nylon 6,6 from the corresponding co-monomers 6-aminohexanoic acid (AHA) and hexamethylene diamine (HMDA), the residues XrLBx, MLC2, and BdSC2 from the xylene extraction (and decarbonation, when required) were treated with refluxing 6 M HCl for 18–24 h to achieve complete depolymerization of the polyamides [26]. At the end of the reaction, the mixtures were allowed to cool and filtered through a 0.22-μm-pore-size PVDF filter. To enable a highly sensitive quantification of the amino monomers, AHA and HMDA (along with amino acids possibly present in the hydrolysate) were derivatized with the fluorophore 5-dimethylaminonaphthalene-1-sulfonyl chloride (dansyl chloride, DNSCl). To this end, 3–5 mL of the acid hydrolysate (as a precisely weighed fraction of the total) was neutralized up to pH 5–6 with 6 N NaOH, then 0.5 mL of the neutralized fraction was added with 1.0 mL concentrated K_2_CO_3_ (16.0 g/L) to precipitate the excess Ca^2+^ that could interfere in the subsequent reaction, and finally with 1.0 mL of DNSCl in acetone (18.5 μmol). After 30 min of stirring at room temperature in the dark, an excess of n-butylamine (5.0 μL, 51⋅μmol) was added to the mixture to quantitatively convert the excess of unreacted DNSCl and the solution was taken to 10 mL in a volumetric flask with a 1:1 acetone/H_2_O mixture. The solution of the derivatized amines was then transferred into a 10-mL volumetric flask and taken to volume with a 1:1 (v/v) water/acetone mixture before reversed-phase HPLC analysis.

### Analytical characterization

#### Pyrolysis-gas chromatography-mass spectrometry (Py-GC/MS)

Analytical pyrolysis was performed with an EGA/PY-3030D® micro-furnace pyrolyzer (Frontier Lab, Japan) coupled to a gas chromatographer 6890 and a single quadrupole Mass Selective Detector 5977B (Agilent Technologies, USA), equipped with an auto-Shot sampler AS-1020E (Frontier Lab, Japan). The chromatographic separation of the pyrolysis products was performed on a fused silica capillary column HP-5MS (5% diphenyl-95% dimethyl-polysiloxane, 30 m × 0.25 mm i.d., 0.25 μm film thickness, J&W Scientific, Agilent Technologies), preceded by 2 m of deactivated fused silica pre-column with an internal diameter of 0.32 mm. Samples were pyrolyzed in single-shot mode at 600 °C for 0.2 min, interface temperature 280 °C. The dried extracts were injected with a split ratio of 20:1, and the GC injector was set at 280 °C. The chromatographic conditions were 40 °C for 6 min, 20 °C/min to 310 °C for 40 min. The helium (purity 99.9995%) gas flow was set in constant flow mode at 1 mL/min. MS parameters: electron impact ionization (EI, 70 eV) in positive mode; ion source temperature 230 °C; scan range 35–550 m/z; quadrupole temperature 150 °C. Agilent Masshunter Workstation (Agilent Technologies) software was used for data analysis, and peak assignment was based on a comparison with libraries of mass spectra (NIST 20, score higher than 80%) and literature data. For the analysis of the DCM extracts, 1 mL of DCM was added to each vial containing the samples dried DCM extracts; then, different volumes of the extract (20–170 μL) were directly dried in the deactivated stainless-steel pyrolysis cup (Py cup) and then weighed with an XS3DU microanalytical scale to obtain about 100 μg of each sample. Regarding the analysis of the xylene extracts, the sample on each filter was quantitatively transferred in the Py cup and weighed with the microanalytical scale before analysis.

Table 1 reports the characteristics and the validation parameters obtained for the five polymers including the pyrolysis products, and the specific m/z signals for each polymer used for integration and quantification. The calibration curves obtained for the polymer feature *r*^2^ in the range 0.9817–0.9994, with LODs and LOQs between 0.007 and 0.05 and between 0.022 and 0.15, respectively. LOD and LOQ were evaluated on the basis of replicate analysis of procedural blanks, and calibration curve parameters. In particular, LOD was calculated as the ratio (average area in the blanks + 3σ)/slope, while LOQ was calculated as the ratio (average area in the blanks + 10σ)/slope, where σ is the standard deviation of the linear regression used to fit the experimental points of the calibration.

**Table 1:** Summary of characteristics and validation parameters for the polymers quantified by Py-GC/MS in the sediment samples: pyrolysis products used for the quantification, m/z used for integration, limit of detection (LOD), limit of quantification (LOQ), and *r*^2^ for the calibration curve. The LOD and LOQ values refer to the amounts introduced in the instrument [28]

Polymer	Selected pyrolysis products	m/z signals	Calibration mass range (µg)	LOD (µg)	LOQ (µg)	*r*^2^
PVC	HCl	36, 38	15–215	0.008	0.026	0.9915
PP	2,4-Dimethyl-1-heptene	70, 126	20–190	0.05	0.15	0.9994
PS	2,4-Diphenyl-1-butene (dimer)	91, 208	0.25–25	0.012	0.042	0.9905
PC	Bisphenol	213	0.50–50	0.007	0.022	0.9817
HDPE	α,ω-Alkenes C_15_–C_25_ (average of the areas^a^)	82	20–190	0.03	0.10	0.9972

^a^11 peaks were integrated for quantifying PE

Phthalate calibration curves were calculated through peak integration of SIM chromatograms, extracting specific m/z signals for each phthalate and internal standard; the m/z adopted are reported in Table 1. For the calibration curves, the peak areas of phthalates were normalized by using DS1 (dibutyl phthalate-3,4,5,6-d4) and DS2(dioctyl phthalate-3,4,5,6-d4) as internal standards (see section “High-performance liquid chromatography (HPLC)”). The absolute values (µg) of the polymers (PVC, PP, PS, PC, HDPE) and plasticizers determined by Py-GC/MS through the calibration curves (Table 1 for the polymers and table S2 in the SI for the phthalates) are related to the micrograms of samples weighed in the Py cup for the analysis. The quantification of the individual polymers and phthalates in the sediment was therefore recalculated based on the actual milligrams of dried extracts obtained after DCM (for PVC,PS, PC, and phthalates) and xylene (for PP and HDPE) extraction, respectively, and to the initial amount of dry sediment, in order to obtain a concentration (µg/kg).

#### High-performance liquid chromatography (HPLC)

Quantitative analysis of the monomers TPA and the dansylated dAHA and dHMDA, obtained by selective depolymerization of PET and of all polyamides and dansylation (derivatization with dansyl chloride) of the latter, was performed with an Agilent 1260 Infinity Binary LC instrument equipped with in series UV-Vis diode array (DAD VL+ 1260/G1315C) and fluorescence (FLD 1260/G1321B) detectors. The UV-Vis detector operating at 242 nm wavelength, a Jones Genesis®AQ reversed-phase column (120 Å, 15 cm length, 4.6 mm internal diameter, particle size 4 μm), and a Jasco column oven set at 30 °C were used for TPA analysis, performed under isocratic conditions at 0.8 mL/min flow rate, and a 40:60 vol% methanol mixture with aqueous trifluoroacetic acid (0.1 wt% in HPLC-grade water) as the eluent. For the quantification of amino monomers AHA and HMDA, a reversed-phase Phenomenex-Aqua C18 column (250 mm × 4.6 mm, 5 μm particle size) with pre-column was used. Injection volume was 20 μL. Elution was performed at a 1.0-mL/min flow rate in gradient mode, using a 2.5 vol% acetic acid and 0.83 vol% freshly distilled trimethylamine aqueous solution (phase A) mixed with acetonitrile (phase B) according to the following program: min 0–20 *A*/*B* = 60/40; min 20–25 gradient to reach *A*/*B* 30/70, then isocratic to min 35; min 35–37 gradient to return to *A*/*B* = 60/40 and then isocratic to min 50. The UV-DAD detector was set at *λ* = 335 nm, while the FLD detector was set at *λ* = 335/522 nm excitation/emission wavelengths.

#### ATR/FTIR spectroscopy

A Thermo Scientific Nicolet iN10 MX spectrometer was used for micro-ATR analysis. The analysis was carried out in ATR mode using a “slide-on” ATR accessory equipped with germanium crystal (IR radiation penetration 0.66 μm at 1000 cm^−1^). Spectra acquisition parameters were 128 scans at a spectral resolution of 4 cm^−1^ in the 700–4000 cm^−1^ spectral range determined by the liquid nitrogen-cooled MCT-A detector. For the analysis of the map in micro-ATR, a 1000 × 1000 μm area was mapped by recording 20 × 20 micro-ATR spectra (total of 400 spectra/map), using an aperture of 200 × 200 μm that determines a spatial resolution of 50 × 50 μm.

## Results

### Characterization of plastics debris with particle size higher than 2.0 mm

The PISA procedure employed in this work for the isolation and polymer-specific analysis of the microplastics in sediments of a nearby marine beach (Lecciona) and marine (off the Serchio River mouth) and coastal lake (Massaciuccoli) benthic sediments allows obtaining mass-based quantitative and qualitative information on the total content of synthetic polymer particles. Size selectivity was ensured by the preliminary sieving to remove (and separately analyze, if appropriate) any inorganic and macroplastic fragments larger than 2 mm, while all information on the size distribution is lost since all synthetic polymer particles are solubilized/depolymerized in their respective procedural step. While the size selectivity is given by the upper size cutoff of the sieve, the mass-based quantitative analysis is typically more sensitive to the presence of large (< 2 mm) microparticles than to nanoparticles (< 1 μm), since the latter must be numerically more abundant by orders or magnitude before contributing significantly to the total mass if large microparticles are present (e.g., a single 10-μm microsphere has the same mass as one million of 100-nm nanospheres).

Objects with size 2 < *d* < 5 mm were found in most of the beach sand samples, but none in the marine benthic sediments, and only a few microfibers with length exceeding 2 mm in the lakebed sediments (figure S2 in the SI). They were collected by sieving the sediment and individually identified by attenuated total reflectance (ATR) infrared spectroscopy*,* as summarized in table S3 in the SI. Some representative images and infrared spectra of the collected microplastic fragments are reported in figures S3-S5 in the SI. As expected, they were all low-density polymers (polyolefins and polystyrene), those that are capable of floating in marine waters and can thus be transported from the source of pollutants input into surface waters to the open sea, eventually ending up in a coastal area. These larger fragments are nearly absent in the swash zone of the beach (foreshore and, to a certain extent, even summer berm) as a result of the cyclical in-out transport due to the action of the waves.

The PISA procedure was then carried out on the dried sediment samples sieved at < 2 mm.

### Analysis of DCM extracts

The first step or the fractionation procedure, as described in sections “Environmental samples” and “Analytical procedures, quality assurance, and quality control (QA/QC) of the quantitative results,” resulted in a set of DCM extracts, likely with various levels of biogenic contaminants (mainly lipids). Since purification procedures could have caused further polymer degradation and/or loss of the most oxidized synthetic polymers, the DCM extracts were not purified but dried, weighed, and then submitted to Py-GC/MS to evaluate the fractional amount of polystyrene and degraded polyolefin contaminants.

**Fig. 2:**
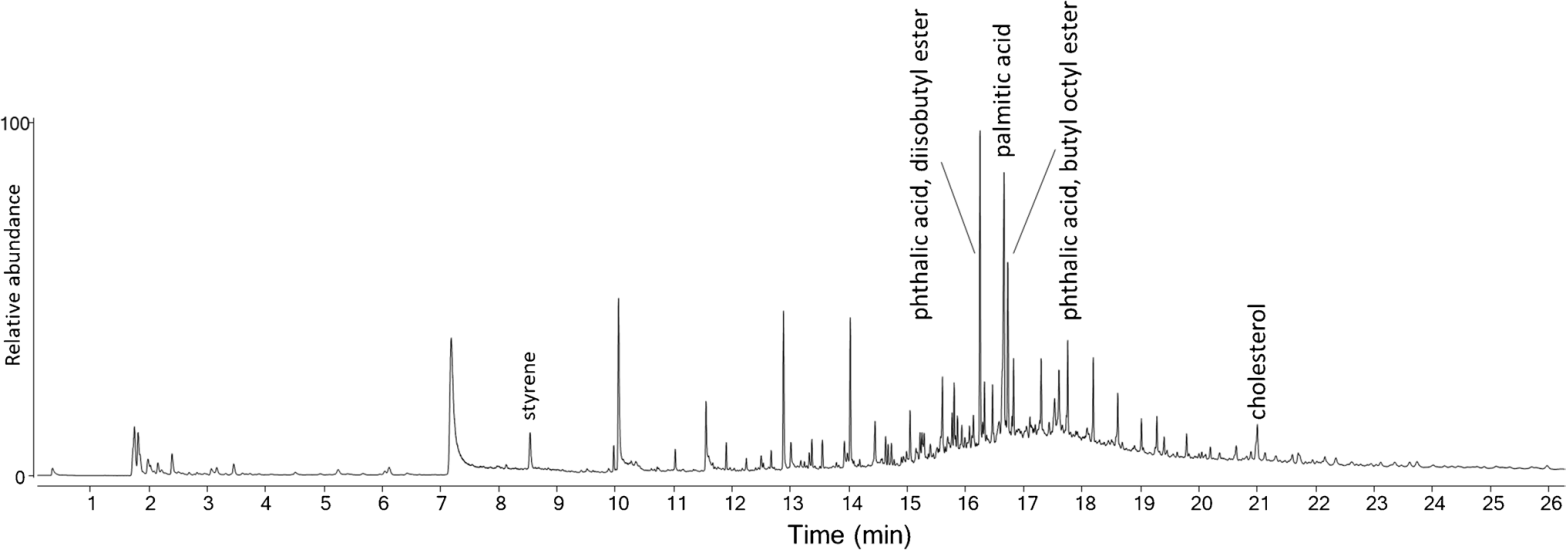
Py-GC/MS chromatogram of the DCM extract of sample LBs3 (DsLBs3)

All the DCM extracts, analyzed by Py-GC/MS, showed the presence of phthalates. The Py-GC/MS chromatogram in Fig. 2 recorded from the DCM extract of sample LBs3 showed the highest concentration. Some samples also showed the presence of sterols (see, for example, figure S6 in the SI). Phthalates are well-known nearly ubiquitous environmental pollutants deriving from their widespread use as plasticizers in some plastics (e.g., PVC), but also in many other formulations (coatings, adhesives, rubbers, etc.); they are easily absorbed by microplastics, the latter acting as environmental concentrators. By using two reference phthalates as internal standards, the total concentration of diethyl phthalate, butyl octyl phthalate, dimethyl phthalate, dibutyl phthalate, benzyl-butyl phthalate, bis(2-ethylhexyl) phthalate, didecan-2-yl phthalate, bis(7-methyloctyl) phthalate, and bis(8-methylnonyl) phthalate could be determined in the 4 < ppb < 2345 range, as detailed in the section “Principal component analysis (PCA) of polymer MP pollution in the Lecciona beach sediment.”

The concentrations of the individual investigated polymers in the DCM extracts of the beach sediment samples (DsLBx in Fig. 1), reported for each sample in table S4 in the SI, were obtained by quantitative Py-GC/MS analysis based on the calibration curves obtained with the reference compounds listed in Table 1. The highest concentrations of these DCM-soluble MPs were found in the dune sector (LBd1-3) of the *Lecciona* beach transects. Among them, the hydrocarbon polymers HDPE (0–864 µg/kg dry sediment), PP (0–720 µg/kg), and PS (0–1139 µg/kg) were largely the most abundant (the broad concentration ranges are indicative of the intrinsic variability among environmental samples). It should be pointed out that HDPE and PP are insoluble in DCM; thus, their extractable fraction must consist of highly oxidized and fragmented oligomers, the high molecular weight fraction being expected as Xy extractables only. Such higher concentrations in the dune sector, the one farther apart from the swash zone, are not surprising. The dune (or any sector far enough from the shore to be outside the swash zone) typically represents an accumulation zone where not only microplastics but also larger plastic debris end up. Once there, they undergo continuous degradation into smaller microplastics that add up to the direct input of MPs from the polluted environment. On the other hand, a different distribution and generally lower concentrations were found for the high-density polymers, with higher amounts in the summer berm and foreshore sectors (0–73 µg/kg of PC and 0–159 µg/kg PVC), presumably because they are less effectively transported to the shore but rather tend to sink into the benthic sediments.

**Fig. 3:**
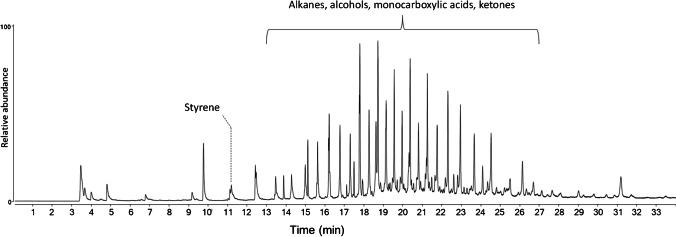
Py-GC/MS chromatogram of the NaOH pre-treated DsMLMe extract

The Py-GC/MS pyrogram of the DCM extract DsML of the sediment from Lake Massaciuccoli not further purified (figure S7 in the SI) showed the presence of a high concentration of sulfur along with alkanes, alcohols, monocarboxylic acids, and ketones apparently of biogenic origin. On the contrary, the pyrogram from the Py-GC/MS analysis on DsMLMe (Fig. 3), obtained after counter-extraction of DsML with 1 M NaOH and isolation of the synthetic polymers by precipitation in CH_3_OH, clearly showed the presence of oligomeric fractions of oxidation and degradation products of polyolefins (in the 14–26-min retention time in the chromatogram) and traces of polystyrene (styrene dimer, even if below the limit of quantification), along with a much smaller amount of the biogenic compound mix already found in DsML. Thus, the counter-extraction with NaOH removed most biogenic compounds masking the presence of polyolefins in the untreated extract.

The Py-GC/MS analysis carried out on the DCM extract of the marine sediment sample DsBdS_mix (figure S8 in the SI), evidently less contaminated by products of biogenic origin, showed the presence of alcohols, alkanes, monocarboxylic acids, and ketones, along with oxidation and depolymerization products of polyolefins and traces of polystyrene below the limit of quantification. In addition to these pyrolysis products, the pyrolytic profile is characterized by the presence of glyceryl triacetate.

### Analysis of xylene extracts

**Fig. 4:**
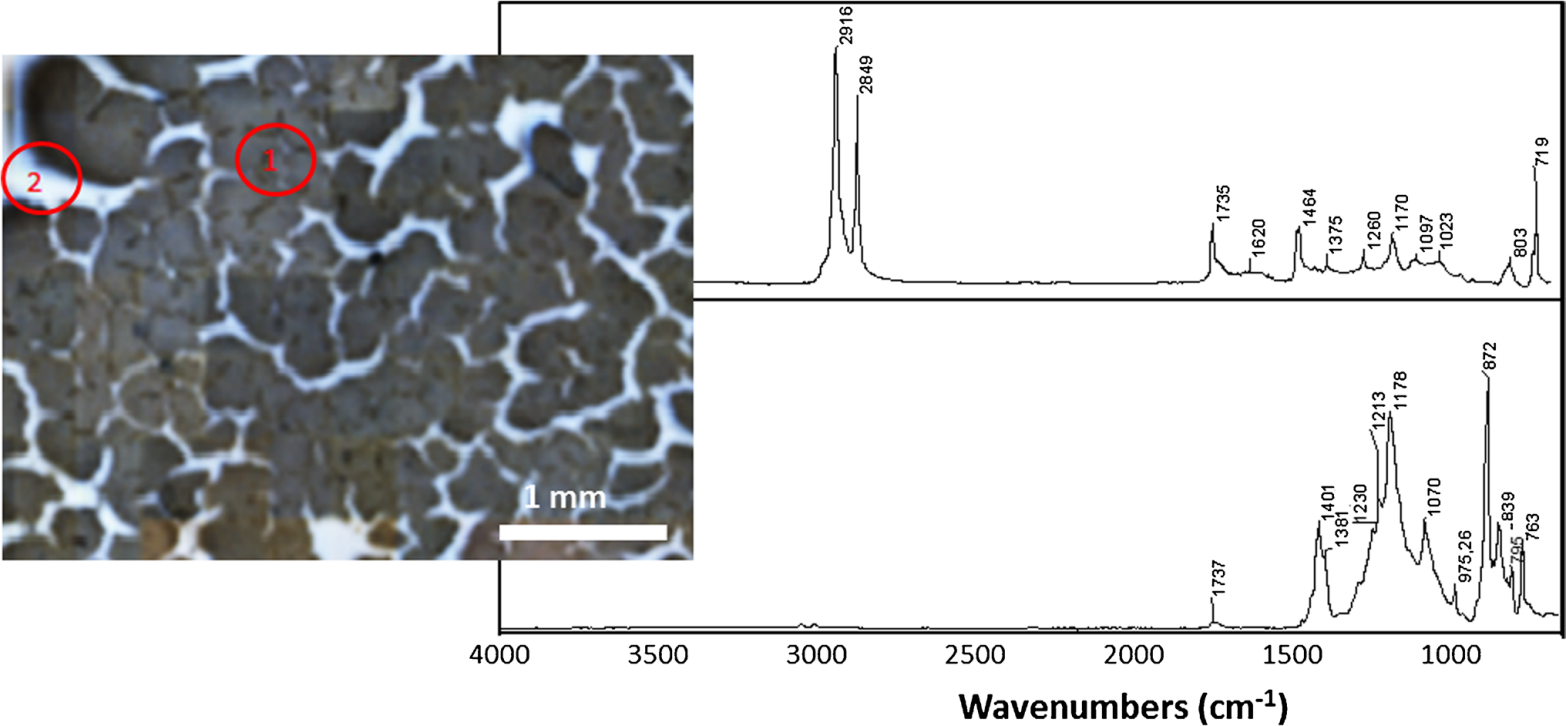
Left: optical micrograph of the solid obtained from the xylene extract XsML after precipitation in MeOH and filtration on a 0.25-μm-pore-size PVDF membrane. Right: micro-ATR FTIR spectra taken from the marked spots in the optical micrographs; above (taken from point 1 of the micrograph), the spectrum of polyethylene, its oxidation shown by the carbonyl absorption at 1735 cm^−1^); below (taken from point 2), the spectrum of PVDF filter in the background

Relatively high and comparable concentrations of polyolefins in the range of 20–40 µg/g sediment (see table S5 in the SI) could be detected and quantified by Py-GC/MS analysis of the xylene extracts from both marine beach (XsLBx in Fig. 1) and lake sediments (XsML; in Fig. 1). As an example, the nearly pure polyethylene precipitate XsML isolated from the lakebed sediment (Fig. 4) gave a corresponding clean Py-GC/MS chromatogram (see figure S9 in the SI). On the contrary, polyolefins were not detected in the Py-GC/MS chromatogram of the traces of solids recovered from the xylene extracts of the marine benthic sediment (figure S10 in the SI).

**Fig. 5:**
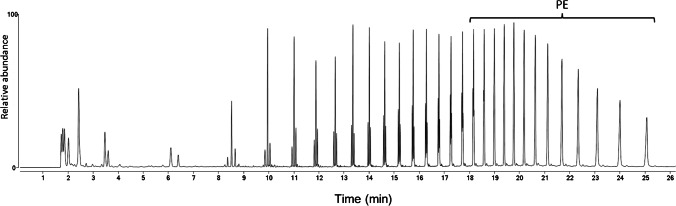
Py-GC/MS chromatogram of the extract of sample LBd2. A detailed list of peak assignments is reported in [24]

Small differences in the weight of Xy extracts were found among the sediments from the four different sectors of the marine beach transects, ranging from 25 to 39 µg/g sediment (see table S5 in the SI; the weighed extracts include some residual biogenic contaminant as shown by Pyr-GC/MS analyses). Unexpectedly, no apparent meaningful correlation was found between such concentrations and the distance from the shoreline in the transect, differently from previous results from a nearby beach [15]. In the Py-GC/MS trace of the xylene extract of LBd2, shown in Fig. 5 as a representative example, the pyrolysis pattern of polyethylene is clearly recognizable, along with peaks due to other oligomeric and low molecular weight biogenic contaminants. A detailed polymer-specific analysis based on the Py-GC/MS results performed on the XsLB samples LBd1, LBd2, and LBd3 of the dune sector gave concentrations (relative to the dry sediment) of < LOD, 1.20, and 1.05 µg/g, respectively, for HDPE, and 0.15, 0.55, and 0.15 µg/g, respectively, PP/sediment.

### PET concentration by TPA analysis after alkaline depolymerization

The concentration of PET was calculated from the concentration of its TPA comonomer in the alkaline hydrolysate of the xylene extraction residues, as detailed in section “Alkaline depolymerization: determination of PET.” For this purpose, 0.5 mL of the neutralized methanolic hydrolysate (as a weighed fraction of the total amount of hydrolysate for quantitation) was mixed with 0.75 mL of 0.1% aqueous H_3_PO_4_ to obtain a final 40/60 vol% MeOH/H_2_O solution. The TPA concentration was then determined by HPLC, and from the TPA concentration, the corresponding PET concentration was calculated (see Table 2).

**Table 2:** PET concentrations (vs. the initial dry sediment) calculated from those of TPA as determined by HPLC

Sampling site	Sediment sample	Sample weight (g)	PET (µg/kg)
Lecciona	LBd1	102.2	^(a)^
	LBd2	101.3	^(a)^
	LBd3	105.5	^(a)^
	LBw1	101.0	20 ^(b)^
	LBw2-i	110.6	^(a)^
	LBw2-ii	100.7	^(a)^
	LBw2-iii	106.2	^(a)^
	LBw3	111.0	^(a)^
	LBs1	103.8	420
	LBs2	99.9	^(a)^
	LBs3	108.1	^(a)^
	LBf1	102.9	^(a)^
	LBf2	112.0	^(a)^
	LBf3	107.1	^(a)^
Lake Massaciuccoli	ML1-3	21.0	770
Serchio River mouth	BdS1-3	27.0	690

^(a)^Concentration, if present, < LOD

^(b)^Concentration detectable (> LOD) but below LOQ

The overall procedure gave the following sensitivity values for the quantification of PET as calculated from the calibrations used for the HPLC analysis of TPA: LOD = 17.3 μg/kg (or ppb, parts per billion), LOQ = 59.0 μg/kg. The obtained results indicate that appreciable concentrations of PET are associated with bottom sediments from both Lake Massaciuccoli and the Serchio River mouth, sensibly because the beach sediments are preferentially subjected to contamination by lower-density, floating microplastics.

### Nylon 6 and Nylon 6,6 concentrations by AHA and HMDA analysis after acid depolymerization

The concentration of the two synthetic polyamides Nylon 6 and Nylon 6,6 was calculated from the concentration of the corresponding DNS-tagged (dansylated) aminated co-monomers AHA and HMDA in the acid hydrolysate of the xylene extraction residues, as detailed in section “Acid depolymerization: determination of Nylon 6 and Nylon 6,6 polyamides.” Due to the peculiar composition of the environmental matrices, reliable results could only be obtained for the sediment samples from Lake Massaciuccoli and the Serchio river mouth. In fact, the beach sediment samples turned out to have a high content of iron minerals that leached out under the highly acidic depolymerization treatment, resulting in iron interference with both the hydrolysis procedure and the subsequent derivatization with DNSCl. Thus, the obtained analytical data for the LB samples were poorly reproducible, as also assessed by running experiments on spiked samples. On the other hand, accurate quantification of the monomers, as also assessed by spiking experiments, was achieved for the two bottom sediment samples, confirming the robustness of the methodology already assessed and validated for much more complex samples such as wastewater treatment plant sludges [26]. The overall procedure gave the following sensitivity values for the quantification of Nylon 6 and Nylon 6,6 as calculated from the calibrations used for the HPLC analysis of the relevant monomers (AHA and HMDA): LOD_Ny6_ = 0.77 μg/kg, LOD_Ny66_ = 0.41 μg/kg, LOQ_Ny6_ = 3.23 μg/kg, LOQ_Ny66_ = 1.37 μg/kg. The concentrations of AHA and HDMA and the calculated concentrations of the corresponding polyamides are reported in Table 3. A representative HPLC trace with the peaks from the dansylated AHA and other amines are reported in figure S11 of the SI.

**Table 3:** HPLC quantitative analysis results for Nylon 6 and Nylon 6,6 MPs in benthic sediments (results for the beach sediments not reported as no reliable data could be obtained)

Sampling site	Sediment sample	Sample weight (g)	6N HCl (mL)	AHA (ng/mL)	Nylon 6 (μg/kg)	HMDA (ng/mL)	Nylon 6,6 (μg/kg)
Lake Massaciuccoli	MLC2	21.0	20	1.6	1.8^(a)^	16.1	29.9
Serchio River mouth	BdSC2	27.0	20	1.3	1.5^(a)^	19.9	28.6

^(a)^Values affected by larger uncertainty as they are LOD < value < LOQ

### Principal component analysis (PCA) of polymer MP pollution in the Lecciona beach sediment

**Fig. 6:**
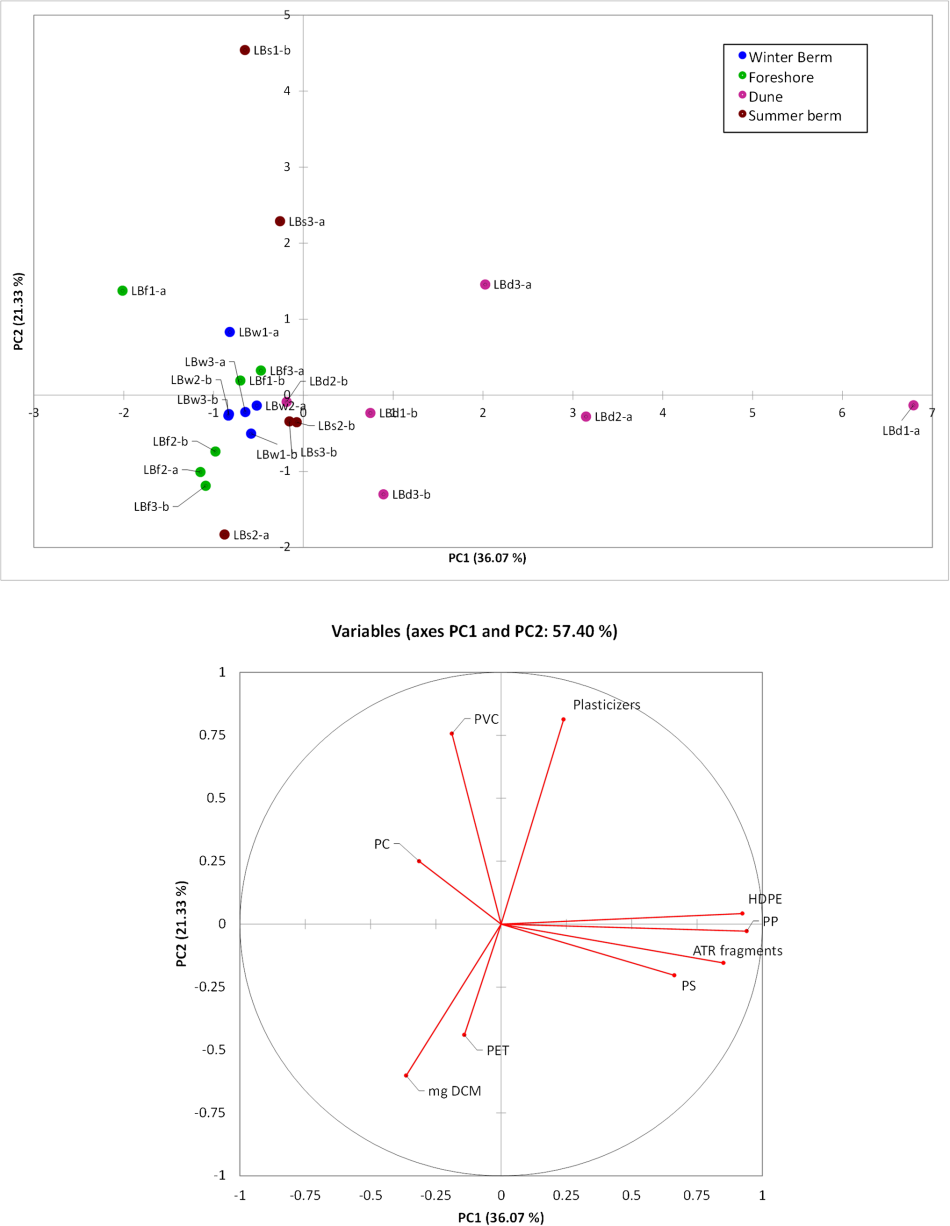
Loading and score plots obtained for the principal component analysis

A more detailed evaluation of the distribution of MPs of different polymeric species was carried out in the case of the 12 samples from three transects of Lecciona beach. When considering the samples as a whole, the concentrations of the investigated polymers, as well as those of the plasticizers, showed high variability as summarized in Table 4. The same data were then processed using principal component analysis (PCA), to assess the possible occurrence of trends in the spatial distribution of the different pollutants on the beach environment (Fig. 6). In particular, the following parameters were considered in the PCA analysis: (i) the concentrations of the different types of MPs and of plasticizers as determined by Py-GC/MS; (ii) the gravimetric data from the dichloromethane solvent extractions; (iii) the number of larger plastic debris obtained by sieving at 2 < d < 5 mm and analyzed by infrared spectroscopy; and (iv) the concentration of PET obtained by alkaline hydrolysis.

**Table 4:** Concentration ranges of synthetic polymers and phthalates polluting the sediment samples of Lecciona beach (the complete dataset with the concentrations for the different polymers detected and quantified by Py-GC/MS in each sample is reported in table S4 in the SI)

Pollutants	Foreshore (µg/kg)	Summer berm (µg/kg)	Winter berm (µg/kg)	Dunes (µg/kg)
Phthalate plasticizers	26–569	206–2340	4–965	36–1230
HDPE	^(a)^	0–202	^(a)^	0–864
PP	^(a)^	0–58	^(a)^	29–720
PS	0–7	0–40	^(a)^	0–1139
PVC	0–82	0–159	0–20	0–12
PC	0–73	0–14	0–28	0–12

^(a)^value < LOD

The sand samples from the summer berm scattered from negative to positive values of PC2. The samples of the summer berm were those characterized by higher abundances of phthalate plasticizes and of the higher-density PC and PVC. In detail, the total amount of plasticizers showed a high variability with values in the range 301–2340 µg/kg for the samples at negative values of PC2, while was characterized by values up to 2340 µg/kg for the sample with the most positive value of PC2. As for the polymers, the PCA result highlight a correlation between PVC, PC, and the plasticizers, all weighting at positive values of PC2: this correlation is probably not incidental since many items made of PC and, in particular, PVC are formulated with high concentrations of phthalate plasticizers.

The three sand samples from the dunes were scattered on the loading plot from negative to positive values of PC1. These samples were characterized by higher amounts of polyolefins (PP and HDPE) and PS MPs as compared to the other sectors of the transects, and by the presence of a comparatively higher number of larger plastic debris (> 2 mm) resulting from the preliminary sieving. Interestingly, a positive correlation was found between the number/nature of such larger plastic debris (mainly PS and HDPE fragments) and the high concentration of the corresponding polymers in the MPs (up to 864 µg/kg of polyolefins and up to 1139 µg/kg for PS in the DCM extracts from the dune sand specimens; see Table 4).

Finally, most of the samples from the winter berm and the foreshore clustered together at negative values for both PC1 and PC2. These samples were characterized by larger amounts of DCM extracts. The loading plot showed a negative correlation between the extracts and the polymers: by analyzing the different pyrolytic profiles of this set of samples, it was possible to highlight that the DCM extracts were characterized by the presence of large fractions of chemical species of biogenic nature rather than deriving from synthetic polymers.

## Discussion

The sediment samples analyzed in this work were collected from nearby marine beach, marine, and coastal lake benthic sediments, representing different potential sinks of MP transport along waterways collecting plastic and microplastic waste from a relatively large, highly anthropized area with environmental pressure from agricultural, urban, and industrial activities. On the other hand, the sampled sites are all enclosed within a natural park area, with lower pressure from local pollution sources and thus an expected main contribution due to the riverine and marine transport.

While the number of samples analyzed was not large enough to grant a detailed and complete picture of microplastic pollution and distribution in the benthic marine and lake sediment and in the unmanaged sandy beach of Lecciona, some interesting features could be highlighted. In general, the types and amounts of MP pollutants show a strict relationship with the different sources (local and land-based for the lake, local and estuarine for the river mouth, estuarine and Mediterranean at large for the marine beach) and the different density and degradation patterns of the different polymers.

The high-density PET and polyamide (nylons) MPs were found at concentrations above their respective LOQ values in both the lakebed and the estuarine seabed hot spots. Such finding suggests a significant contribution from the riverine and canalized surface waters collecting urban (treated) wastewaters and waters from the densely anthropized area of Lucca province (through the Serchio River) and the Arno River aquifer that includes most northern Tuscany. In addition, the lakebed sediment was found to contain a very large amount of highly oxidized polyethylene (about 200 mg per kg, or 200 ppm), with a likely main contribution from MPs leached from not properly disposed of, and progressively degrading, plastic mulching films in agricultural practices surrounding the lake and outside the park area. The presence of a relatively large amount of PET and nylons (about 770 and 30 µg/kg of dry sediment, respectively) is presumably due to the uncaptured textile fibers in the treated waters from a small wastewater treatment plant that discharges into a canal eventually entering Lake Massaciuccoli. Indeed, the lake sediments can be considered as the ultimate sink for MPs entering a lake that is only loosely connected to the nearby marine environment through a canal (Burlamacca) with a lock to prevent saltwater from entering the lake, only occasionally opened to discharge abundant rainfall. In the benthic sediment of the shallow BdS, as expected, only traces of PS and of highly degraded polyolefins but significant amounts of the higher-density PET and polyamides were found, likely as a result of the entry of the Serchio River waters with their load of textile fibers that, given their relatively high density, are likely to reach the seabed within a relatively short range once in the marine environment.

The sandy marine beach of Lecciona, lying along the 4-km-wide stretch of land separating Lake Massaciuccoli from the sea, and midway between the mouth of Serchio River (6 km to the south) and the harbor of the touristic town of Viareggio connected to the lake through the Burlamacca canal, receives plastic debris mostly as floating material carried by marine currents. Such debris, typically based on low-density polyolefins and PS both as MPs and as larger plastic items, is prone to undergo progressive environmental (photo-oxidative) degradation [29]. However, also higher-density polyester (PET) and nylon microfibers as well as other high-density MPs (PVC, PC) could reach the beach from the nearby estuarine waters. The concentrations of the individual polymers in the DCM-extractable MPs (listed in detail in table S4 of the SI) indicate the dune as an accumulation zone for PS (up to 1138 µg/kg) and for highly oxidized polyolefins. The high tide and storm berm or, even more so and if present, the dune zone, have been reported as accumulation zones by several authors [30, 31], although opposite trends have been found in the case of polymer pellets (size > 1 mm) [32], indicating the relevance of the particle size and shape in determining the distribution across the beach. Even uncorrelated data as a result of specific local environmental conditions (wind, currents, estuarine proximity) [33] or possibly of inconsistencies deriving from, e.g., the comparison of number (particle) vs. total mass (our approach) quantifications [34] indicate that multiple factors may affect the distribution of particles in sandy beaches. In our case, the presence of PS is presumably due to the contributions from larger fragments of expanded PS (a very frequently spotted kind of debris from packaging and also a material extensively used in fishery) and highly oxidized polyolefin MPs generated by slowly degrading larger debris, as the latter are not removed by the swash action of the waves as effectively as in the sectors closer to the shoreline. The not negligible concentrations of higher-density MPs, particularly in the zones closer to the shoreline, may be the result of marine transport from the nearby Serchio and Arno rivers and of waste abandoned on this unmanaged natural beach by passers-by and beach tourists. Relatively high concentrations of highly oxidized polyolefin MPs (up to 864 µg/kg of dry sediment detected as DCM extractables) were also found in the beach dune sector, likely as a result of progressive degradation of larger plastic items in analogy with the case of PS MPs.

The PCA analysis on the Lecciona beach sediment samples highlighted a correlation between the polymer type and the loading of phthalates. The more polar PVC and PC were more heavily contaminated with phthalates than the apolar hydrocarbon polymers (PE, PP, PS). In addition, a positive correlation was found between the presence of larger PS and HDPE debris (2 mm < *d* < 5 mm) in the accumulation zone of the beach dune, and a higher total mass of MPs (< 2 mm) of the same polymer type in the given sample. Such correlation suggests that larger fragments exposed to relatively harsh photo-aging conditions such as those typical of a dune environment (very hot and with intense solar irradiation) are effective and continuous sources of MPs as a result of ongoing degradation. The concentration of plasticizers in the sediment samples collected from the summer berm and the dune were too high to be exclusively associated with the MP contaminants in the samples alone; moreover, some phthalates are no longer used in polymer formulations (e.g., in PVC). Therefore, such relatively high concentrations of phthalates are likely the result of the MPs acting also as absorbers, and not only as release sources, of phthalates from polluted environments during their transport along the waterways and in the oceans. The presence of phthalates could also be linked to transport phenomena through marine aerosol that may travel hundreds of meters from the shoreline, with a mechanism similar to the one that involves surfactants; indeed, the presence of surfactants (i.e., their characteristic pyrolysis products) was detected in the Py-GC/MS analysis of the DCM extracts of the dune sediments. Already in the 1960s and 1970s of the last century, studies carried out in the area of San Rossore had highlighted the key role of surfactants in promoting airborne transport of nonvolatile matter through marine aerosol; their concentration was found to be higher in the marine aerosol, compared to the surface layer of seawater [35–37]. Because of that, considering the plasticizers as trackers of microplastics, as reported in some papers, is likely to lead to inaccurate quantitative results and should be avoided.

Finally, surprisingly low concentrations of less degraded polyolefins were found as xylene extractables in the marine beach sediments, with sector-averaged mass concentrations ranging from 25 to 39 µg/kg (see table S5 in the SI). In particular, the lack of significant differences among polyolefin MP concentrations in the four sectors of the transects was quite unexpected, since a previous investigation carried out in a nearby beach by the same research team and with the same analytical approach had highlighted an uneven distribution along the transect, with higher concentrations found again in the dune sector [15]. These apparently inconsistent results have prompted further investigations with new results that will be elaborated and presented in a forthcoming paper.

## Supplementary Information

Below is the link to the electronic supplementary material.

Supplementary file1 (DOCX 14.3 MB)
