# Supplementary material for: Microplastic pollution in the sediments of interconnected lakebed, seabed, and seashore aquatic environments: polymer-specific total mass through the multianalytical “PISA” procedure
Source: Anal Bioanal Chem. 2023 Apr 18;415(15):2921–36. doi: 10.1007/s00216-023-04664-0 (PMC10285023; doi:10.1007/s00216-023-04664-0)
Supplement: Supplementary file 1 — Supplementary file1 (DOCX 14.3 MB) [file 216_2023_4664_MOESM1_ESM.docx]

**Supplementary Information**

*Analytical and Bioanalytical Chemistry*

**Microplastic pollution in the sediments of interconnected lakebed, seabed, and seashore aquatic environments: polymer-specific total mass through the multianalytical “PISA” procedure**

Andrea Corti^1,2^, Jacopo La Nasa^1,2^, Greta Biale^1^, Alessio Ceccarini^1,2^, Antonella Manariti^1,2^, Filippo Petri^1^, Francesca Modugno^1,2^, Valter Castelvetro^1,2,^*

^1^ Department of Chemistry and Industrial Chemistry, University of Pisa, 56124 Pisa, Italy

^2^ CISUP - Center for the Integration of Scientific Instruments of the University of Pisa, University of Pisa, 56124 Pisa, Italy

Corresponding author: Valter Castelvetro; *valter.castelvetro@unipi.it* ORCID: 0000-0002-3302-7037


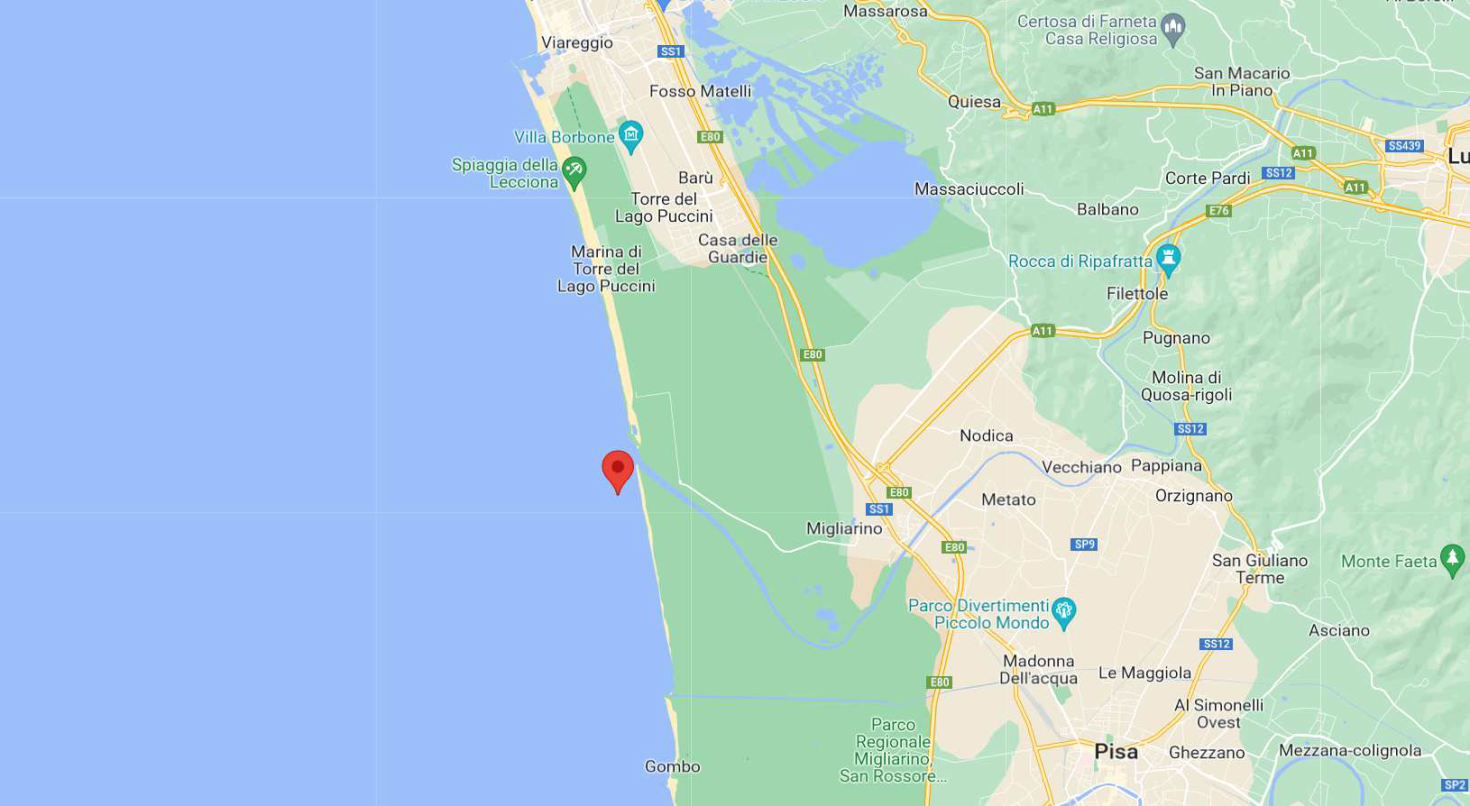

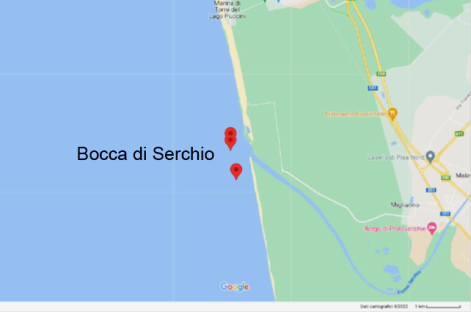


**Serchio river→**

**🡨Lecciona beach**

**1 km**

**↑**

**Massaciuccoli lake**

**1**

**2**

**3**

**San Rossore Regional Park**


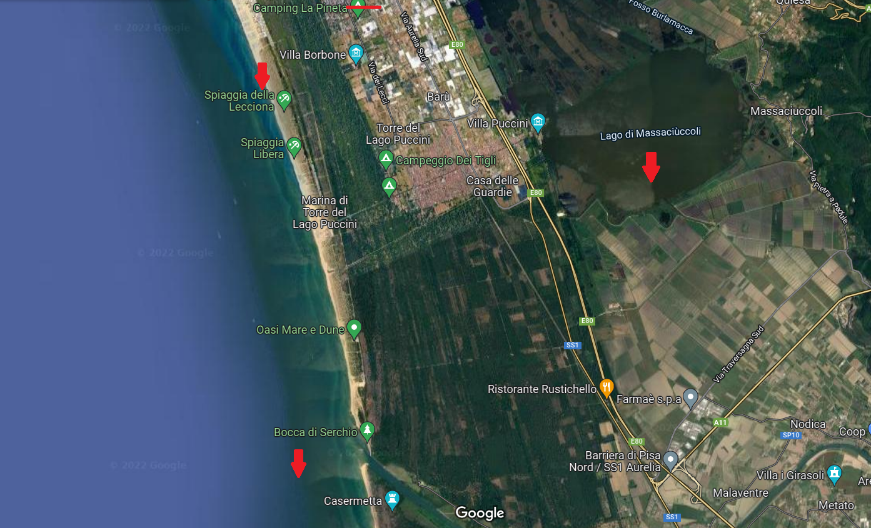


**Figure S1** Map of the sampling site (red arrows) within the San Rossore-Migliarino-Massacciuccoli natural park. [Map data above: ©2019 Google Maps; earth map below: ©2023 CNES /Airbus,Landsat/Copernicus,Maxar Technologies,Dati cartografici ©2023]

In the samples from Lake Massaciuccoli, two fibers were isolated, shown in **Figure S2**. The blue fiber, on the left was identified via ATR as acrylic fiber, while the red one, on the right showed an ATR spectrum coinciding with that of partially degraded cellulose acetate.

Figure S3 shows some representative fragments isolated from the samples of the Lecciona beach, while in Figure S4 and Figure S5 are reported the relative ATR spectra.


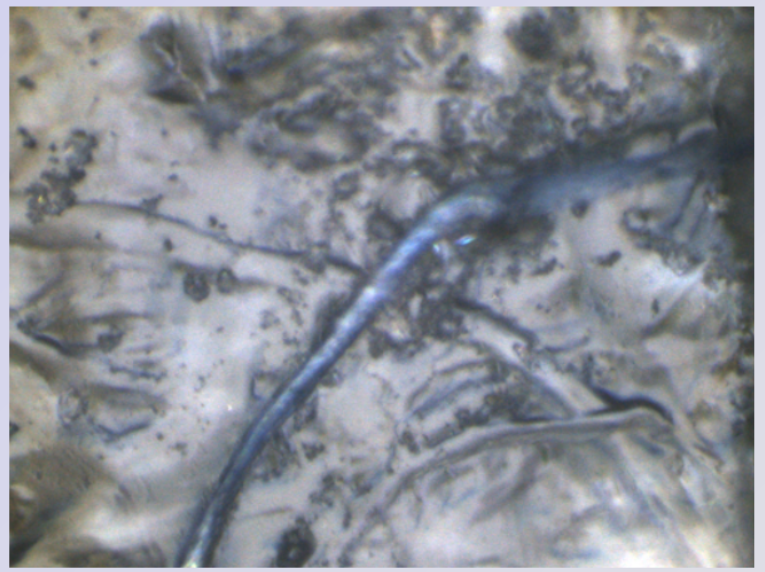

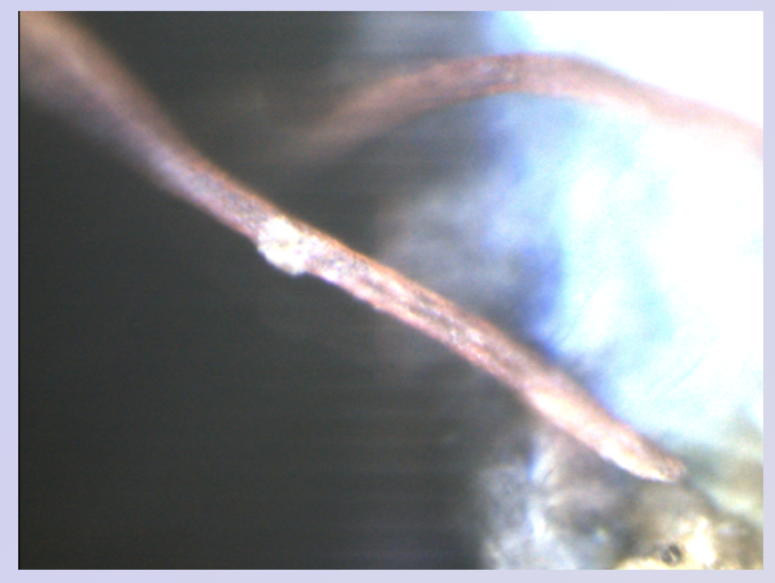


**Figure S2** Fibers isolated by density separation from Lake Massaciuccoli sediment (picture frame witdh = 1 mm).

| 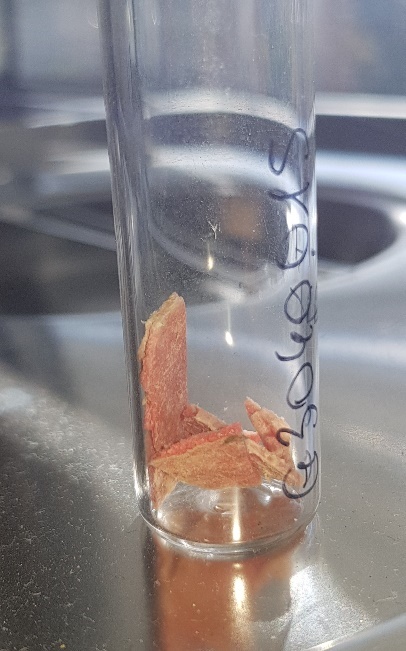 | 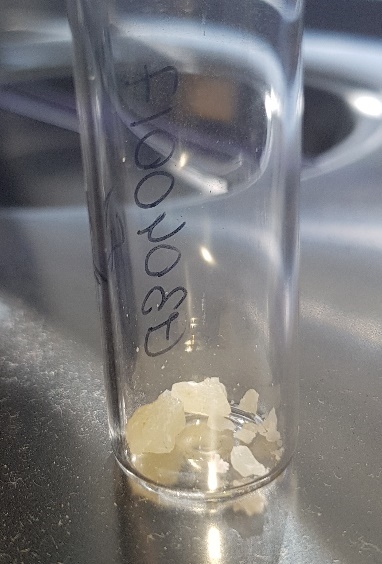 | 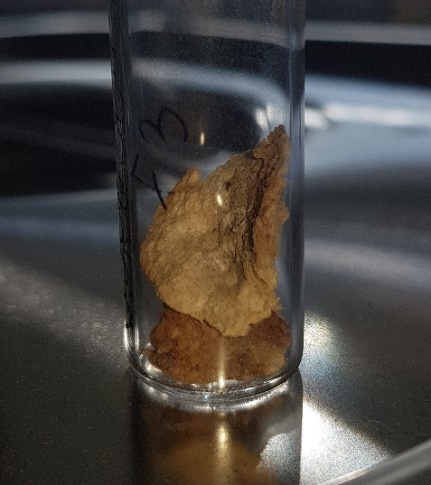 |
| --- | --- | --- |
| (a) | (b) | (c) |

**Figure S3** Photographs of fragments placed in 5 mL vials a) HDPE; b) PP; c) PS; present in samples taken from Lecciona beach.


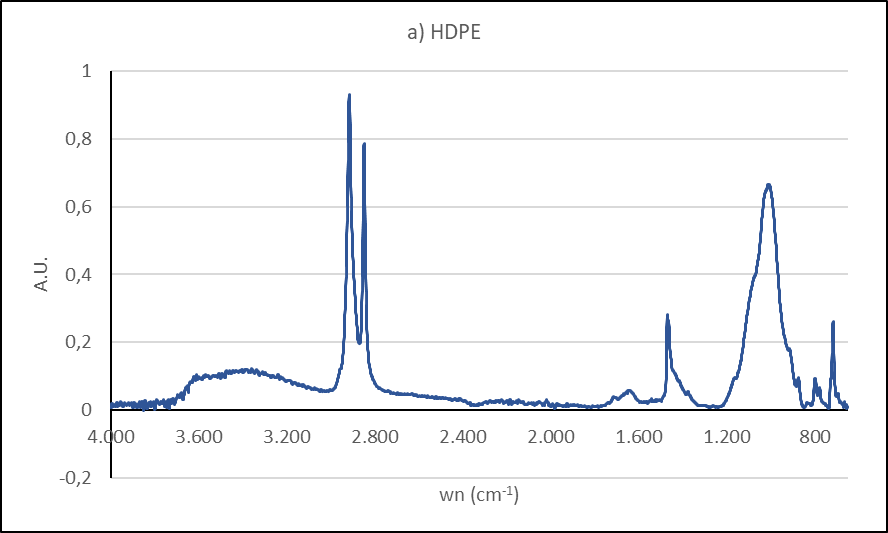

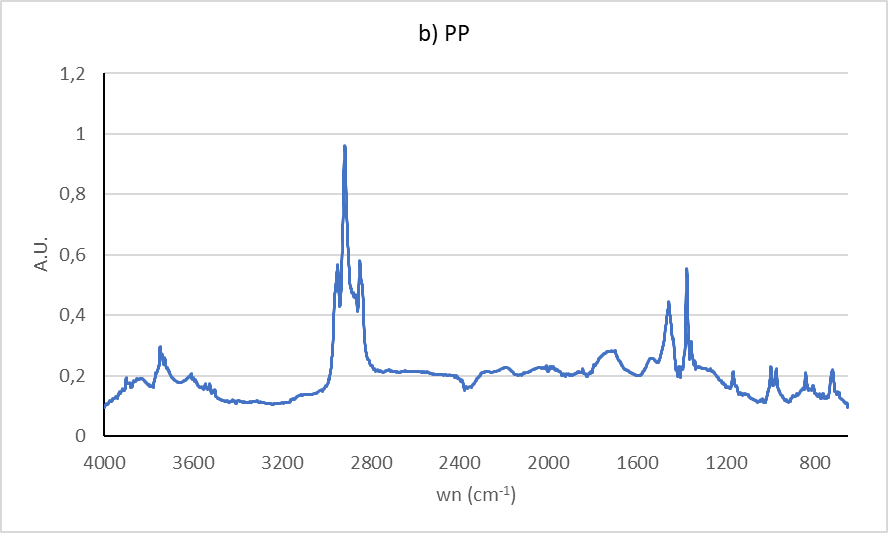


**Figure S4** ATR spectra of fragments LBd2-f1 (a) and LBd1-f1 (b) from Lecciona dune sand samples.


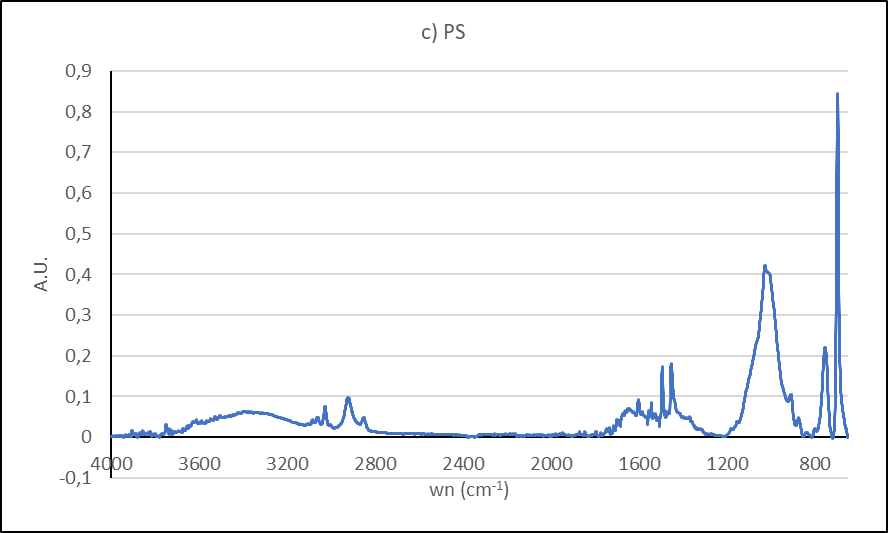


**Figure S5** ATR spectrum of fragment LBd1-f2 from Lecciona dune sand samples.

**Figure S6** Py-GC/MS chromatogram of the DCM extract of sample LBf1, showing the presence of biogenic sterols.

**Figure S7** Py-GC/MS chromatogram of the DCM extract, DsML.

**Figure S8** Py-GC/MS chromatogram of the DCM extract of the marine sediment sample, DsBdS_mix.

**Figure S9** Py-GC-MS chromatogram of the Xy extract XsML.

**Figure S10** Py-GC-MS chromatogram of the xylene extract from the benthic marine sediment XsBdS, showing only the presence of non-specific pyrolysis markers and of bis(2-ethylhexyl)isophthalate, a common plasticizer.

**Figure S11** Chromatographic separation of the dansylated derivatives of AHA and of n-butylamine (reagent used to convert the excess dansyl chloride at the end of the derivatization reaction)

**Table S1** List of the sampling sites and amount of sampled sediment

| **Sediment sample** | | **Geolocation** | **Sampling zone** | **Wet Weight**  **(g)** | **Dry Weight**  **(g)** | **Water Loss**  **(%)** |
| --- | --- | --- | --- | --- | --- | --- |
| **Lecciona** | LBd1 | 43.836859 - 10.243384 | dune | 1958.1 | 1903.5 | 2.8 |
|  | LBd2 | 43.831546 10.251203 | dune | 1894.9 | 1818.6 | 4.0 |
|  | LBd3 | 43.830445 10.251627 | dune | 1941.0 | 1892.3 | 2.5 |
|  | LBw1 | 43.850859 - 10.243384 | winter berm | 1971.0 | 1921.0 | 2.5 |
|  | LBw2 | 43.831447 - 10.250860 | winter berm | 2219.1 | 2141.8 | 3.5 |
|  | LBw3 | 43830329 - 10.251334 | winter berm | 1940.2 | 1880.9 | 3.1 |
|  | LBs1 | 43.834454 - 10.253382 | summer berm | 1364.4 | 1299.7 | 4.7 |
|  | LBs2 | 43.831381 - 10.250629 | summer berm | 1955.2 | 1883.4 | 3.7 |
|  | LBs3 | 43.830316 - 10.251195 | summer berm | 1943.0 | 1884.0 | 3.0 |
|  | LBf1 | 43.849122 - 10.245527 | Foreshore | 1918.6 | 1863.8 | 2.9 |
|  | LBf2 | 43.831391 - 10.250557 | Foreshore | 1646.8 | 1545.9 | 6.1 |
|  | LBf3 | 43.830319 - 10.251068 | Foreshore | 1625.5 | 1405.9 | 13.5 |
| **Massacciuccoli Lake** | ML1 | 43.823389 - 10.333278 | - | 474.3 | 215.6 | 54.5 |
|  | ML2 |  |  | 486.1 | 215.1 | 55.8 |
|  | ML3 |  |  | 454.5 | 210.7 | 53.6 |
| **River Serchio estuarine platform** | BdS1 | 43.783800 - 10.260767 | - | 859.3 | 533.7 | 37.9 |
|  | BdS2 | 43.782300 - 10.260617 |  | 963.1 | 601.9 | 37.5 |
|  | BdS3 | 43.774367 - 10.262600 |  | 739.2 | 465.5 | 37.0 |

**Table S2** Summary of characteristics and validation parameters obtained for phthalates: molecular weight (MW), m/z used for SIM acquisition in the Py-GC/MS analysis, limit of detection (LOD), limit of quantification (LOQ), and r^2^ for the calibration curve [23]. The concentration of the phthalate plasticizers were in the range 0.5-15 µg

| Plasticizer | MW  (Da) | m/z used for SIM acquisition | LOD  (µg) | LOQ  (µg) | r^2^ |
| --- | --- | --- | --- | --- | --- |
| dimethyl phthalate | 194 | 163, 194 | 0.007 | 0.024 | 0.993 |
| dibutyl phthalate | 278 | 149, 223 | 0.022 | 0.073 | 1.000 |
| benzylbutyl phthalate | 312 | 149, 206, 238 | 0.002 | 0.008 | 0.998 |
| bis(2-ethylhexyl) phthalate | 390 | 149, 279 | 0.001 | 0.004 | 0.999 |
| bis(7-methyloctyl) phthalate | 418 | 149, 293 | 0.008 | 0.027 | 1.000 |
| bis(8-methylnonyl) phthalate | 446 | 149, 307 | 0.005 | 0.017 | 0.999 |

**Table S3** Number, weight, and identification of fragments larger than 2.0 mm sieved off the sediment samples of Lecciona beach.

| Sampling sector | Sediment sample | Number of fragments > 2 mm | Total weight ^(a)^  (mg) | Fragment | Polymer type |
| --- | --- | --- | --- | --- | --- |
| Dune | LBd1 | 2 | 99.0 | f1 | PP |
|  |  |  |  | f2 | PS |
|  | LBd2 | 13 | 709.8 | f1 | HDPE |
|  |  |  |  | f2 | HDPE |
|  |  |  |  | f3 | PS |
|  |  |  |  | f4 | HDPE |
|  |  |  |  | f5 | HDPE |
|  |  |  |  | f6 | HDPE |
|  |  |  |  | f7 | PP |
|  |  |  |  | f8 | HDPE |
|  |  |  |  | f9 | PS |
|  |  |  |  | f10 | PS |
|  |  |  |  | f11 | PS |
|  |  |  |  | f12 | PS |
|  |  |  |  | f13 | PS |
|  | LBd3 | 4 | 170.5 | f1 | HDPE |
|  |  |  |  | f2 | PS |
|  |  |  |  | f3 | HDPE |
|  |  |  |  | f4 | PP |
| Winter berm | LBw2 | 1 | 5.7 | f1 | HDPE |
|  | LBw3 | 1 | 4.8 | f1 | HDPE |
| Summer berm | LBs1 | 5 | 82.9 | f1 | HDPE |
| Foreshore | LBf1 | 1 | 5.7 | f1 | n.d. |

(a) Total weight of fragments > 2mm

**Table S4** Concentrations of synthetic polymers (MPs) and low molecular weight compounds (total phthalates) given as µg /kg dry sediment, as determined by Py/GC-MS for each chemical species in the DCM extracts from the sediments samples DsLBx from Lecciona beach (a and b stand for repeated analyses on two fractions of the same sediment taken after mechanical homogenization).

| Analyte | Dune | | | | | | Winter berm | | | | | | Summer berm | | | | | | Foreshore | | | | | |
| --- | --- | --- | --- | --- | --- | --- | --- | --- | --- | --- | --- | --- | --- | --- | --- | --- | --- | --- | --- | --- | --- | --- | --- | --- |
|  | Lbd1  a | Lbd2  a | Lbd3  a | Lbd1  b | Lbd2  b | Lbd3  b | Lbw1  a | Lbw2  a | Lbw3  a | Lbw1  b | Lbw2  b | Lbw3  b | Lbs1  a | Lbs2  a | Lbs3  a | Lbs1  b | Lbs2  b | Lbs3  b | Lbf1  a | Lbf2  a | Lbf3  a | Lbf1  b | Lbf2  b | Lbf3  b |
| Phthalates | 1228 | 563 | 1861 | 62 | 749 | 36 | 965 | 587 | 81 | 4 | 749 | 779 | 301 | 206 | 2321 | 2340 | 589 | 563 | 570 | 143 | 340 | 96 | 478 | 26 |
| PS | 803 | 475 | 0 | 14 | 0 | 1139 | 0 | 0 | 0 | 0 | 0 | 0 | 40 | 22 | 0 | 0 | 10 | 8 | 0 | 0 | 0 | 0 | 0 | 7 |
| PC | 0 | 12 | 0 | 0 | 0 | 7 | 28 | 0 | 5 | 1 | 3 | 5 | 13 | 13 | 14 | 11 | 0 | 7 | 73 | 11 | 0 | 18 | 0 | 4 |
| PVC | 0 | 10 | 12 | 7 | 7 | 0 | 20 | 0 | 11 | 0 | 5 | 0 | 0 | 24 | 13 | 159 | 4 | 0 | 82 | 0 | 9 | 0 | 7 | 0 |
| HDPE | 864 | 288 | 432 | 115 | 0 | 0 | 0 | 0 | 0 | 0 | 0 | 0 | 0 | 0 | 0 | 0 | 202 | 202 | 0 | 0 | 0 | 0 | 0 | 0 |
| PP | 720 | 288 | 91 | 48 | 82 | 29 | 0 | 0 | 0 | 0 | 0 | 0 | 0 | 0 | 0 | 0 | 58 | 58 | 0 | 0 | 0 | 0 | 0 | 0 |

**Table S5** Gravimetric analysis of xylene extracts.

| Site | Sample | Weight  (g) | Xylene extract  (mg) | Xylene extract  (µg/g) | Average Xy extract  (µg/g) |
| --- | --- | --- | --- | --- | --- |
| Lecciona | LBd1 | 36.98 | 1.4 | 37.86 | 32.9 |
|  | LBd2 | 38.38 | 1.4 | 36.48 |  |
|  | LBd3 | 36.82 | 0.9 | 24.44 |  |
|  | LBw1 | 40.20 | 2.9 | 72.14 | 35.1 |
|  | LBw2-i | 40.50 | 1.0 | 24.69 |  |
|  | LBw2-ii | 44.10 | 1.8 | 40.82 |  |
|  | LBw2-iii | 36.97 | 1.1 | 29.75 |  |
|  | LBw3 | 36.34 | 0.3 | 8.26 |  |
|  | LBs1 | 33.61 | 0.0 | 0.00 | 25.9 |
|  | LBs2 | 38.42 | 1.4 | 36.44 |  |
|  | LBs3 | 33.85 | 1.4 | 41.36 |  |
|  | LBf1 | 35.26 | 0.3 | 8.51 | 39.1 |
|  | LBf2 | 36.13 | 0.8 | 22.14 |  |
|  | LBf3 | 41.54 | 3.6 | 86.66 |  |
| River Serchio Mouth | BdSmix | 64.25 | 0 ^(a)^ | n.d. | n.a. |
| Massacciuccoli lake | MLmix | 79.54 | 2.4 | 19.35 | n.a. |

^(a)^ Within the 0.1 mg instrumental error
